# Aged Parents

**Published:** 1898-09

**Authors:** 


### Aged Parents.

A woman in Toronto who is over sixty years of age gave birth on January 21st, to a baby girl. Her husband, to whom she was married seven years ago, is seventy-eight years of age. The mother has been married twice, and this is her twenty-second child. The day after this, not to be outdone by any foreigner, a woman sixty-five years old, living in Oklahoma, also gave birth to a baby girl. This mother has also a number of other children ranging in age from thirty-five to forty-five years. Some of the male sex have, as might naturally be supposed, better records than this. A Tyrolese gentleman named Parravicini, is reported to have married at the age of eighty-two years, and to have been the father of seven children, the last of whom was posthumous, his father having died at the age of one hundred and four years. Juba, king of Mauritania, is believed to have died at the age of ninety-one years, leaving a posthumous child.—*N. Y. Med. Record.*

